# Ultra-broadband, lithography-free, omnidirectional, and polarization-insensitive perfect absorber

**DOI:** 10.1038/s41598-021-84889-0

**Published:** 2021-03-04

**Authors:** Tse-An Chen, Meng-Ju Yub, Yu-Jung Lu, Ta-Jen Yen

**Affiliations:** 1 Department of Materials Science and Engineering, National Tsing Hua University, No. 101, Section 2, Kuang-Fu Road, Hsinchu, 30013 Taiwan, ROC; 2 Research Center for Applied Sciences, Academia Sinica, 128 Sec. 2, Academia Rd., Nankang, Taipei City, Taiwan

**Keywords:** Engineering, Optics and photonics

## Abstract

Perfect absorbers (PAs) at near infrared allow various applications such as biosensors, nonlinear optics, color filters, thermal emitters and so on. These PAs, enabled by plasmonic resonance, are typically powerful and compact, but confront inherent challenges of narrow bandwidth, polarization dependence, and limited incident angles as well as requires using expensive lithographic process, which limit their practical applications and mass production. In this work, we demonstrate a non-resonant PA that is comprised of six continuous layers of magnesium fluoride (MgF_2_) and chromium (Cr) in turns. Our device absorbs more than 90% of light in a broad range of 900–1900 nm. In addition, such a planar design is lithography-free, certainly independent with polarization, and presents a further advantage of wide incidence up to 70°. The measured performance of our optimized PA agrees well with analytical calculations of transfer matrix method (TMM) and numerical simulations of finite element method, and can be readily implemented for practical applications.

## Introduction

Recently electromagnetic absorbers realized by plasmonic resonance, termed as perfect absorbers (PAs), have been attracting increasing attention, mainly because these PAs promise great absorbance and compact sizes simultaneously. By efficiently suppressing the reflectance and the transmittance of the undesirable electromagnetic radiations, there demonstrated a wide range of potential applications, such as stealth technologies^1^, communication antennas^2,3^, radars^4^, thermal emitters^5,6^, sensors^7–9^, photodetectors^10–12^, and so on. Conventionally, such PAs adopted a common design of resonant metal-dielectric-metal (MDM) structure. In this structure, a patterned top metal layer provides the excitation of not only electric but also magnetic resonances, which couple to match the surface impedance and thus reduce the reflectance^9,13–15^. Next, a dielectric layer, sandwiched in the middle, allows a Fabry–Perot cavity mode to boost the absorption efficiency. Finally, a bottom metal layer that is typically a continuous ground, blocks the propagation of electromagnetic waves, efficiently terminating the transmittance. In addition, to obtain a stronger plasmonic resonance in these PAs, usually researches employed noble metals, such as silver (Ag) and gold (Au), because of their low loss properties.

These PAs are powerful and compact; however, their performance remains unsatisfactory. The hurdle is obviously associated with the resonance nature, such that these PAs suffer from intrinsic limitations of narrow bandwidths, a low tolerance for incidence angle variations, and a narrow range of working polarization angles. More recently, several strategies have been proposed to improve the absorption performance of the resonant PAs. For example, a straightforward solution is to combine various sub-wavelength patterns with different shapes and/or dimensions to broaden the absorption bandwidth^16–22^. Yet, those sub-wavelength patterns demand lithographic fabrication, which are costly and cumbersome, especially for the applications of large surface areas and high working frequencies (i.e., tiny feature sizes). Therefore, the fabrication of PAs without the use of EB lithography, such as multilayer deposition^23–31^, oblique-angle deposition^32,33^, and chemical processes^34–36^, has been proposed to overcome the drawbacks of structural metamaterials. Among those methods, the multilayer deposition method outperforms others because the multilayer deposition method is not only more feasible, but also achieves the larger bandwidth and higher incident angle tolerance simultaneously. Different combinations of metals and insulators have been used to enhance ultra-broadband light absorption. Increasing the number of paired layers is an efficiency way to expand the absorption bandwidth and absorbance. However, this requires the deposition of multiple layers which makes sample fabrication more difficult.

As a consequence, in this work we presented a superior absorber in near-infrared regime, to meet the critical challenges aforementioned. The demonstrated absorber was a non-resonant design that is comprised of six continuous layers of magnesium fluoride (MgF_2_) and chromium (Cr) in turns. The materials adopted here were economic, in contrast to the noble metals used in conventional PAs. As for the structural design and realization, firstly, we analytically employed the transfer matrix method (TMM) to design and to optimize the absorption spectra of the electromagnetic absorber, with respect to various layer thicknesses. Next, by using a commercial solver (Comsol Multiphysics 5.3a) to conduct numerical simulation^37^, we confirmed our device achieving great absorbance beyond 90% over a broad range of 900–1900 nm. Such great and ultra-broadband absorption was independent with polarization angles, and also validated with the wide oblique incidence up to 70°. In addition, we further scrutinized the mechanism and contribution of designed each layer. Finally, to fabricate this non-resonant PA, it only required a facile thin-film deposition process that was totally free from using costly and cumbersome lithography. The corresponding experimental results, analytical calculation and numerical simulation were all in good agreement. To the best of our knowledge, this non-resonant PA shows the broadest absorption range. In addition, the realization of this PA only requires one-step of deposition process, free from the use of lithography. With the advantages of superior performance and facile fabrication process, this non-resonant PA can be readily employed for a wide range of practical applications.

## Analytical calculation and numerical calculation

**Figure 1:**
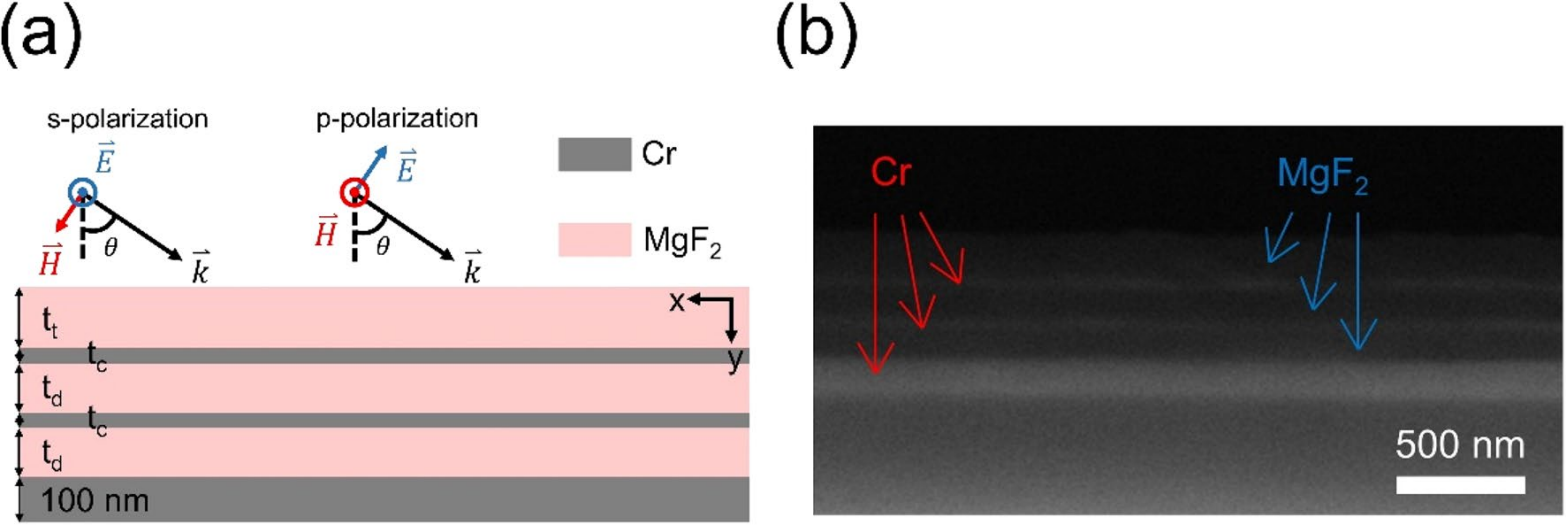
Schematic illustration of the fabricated structure and showing its (**a**) excitation configuration and (**b**) the cross section of SEM image. In the optimal configuration, t_t_ = 230 nm, t_c_ = 7 nm, and t_d_ = 180 nm.

The non-resonant PA at NIR regime is comprised of MgF_2_ and Cr, six thin layers pile-stacking alternatively, as shown in Fig. 1. There appear two reasons to choose MgF_2_ as a dielectric material. One is based its low real part of complex permittivity, which facilitates to suppress the reflectance to the air (see the Sect. 1 of Supplementary Information). As for the reasons of selecting Cr as the metallic material, its imaginary part of complex permittivity is much higher than other metals, such that Cr can absorb light efficiently (see the Figure S2 in Sect. 2 of Supplementary Information). Note that an absorber converts electromagnetic energy into heat and thus, the materials used to construct the electromagnetic absorbers should withstand elevated temperatures. MgF_2_ and Cr possess high melting points of 1263 and 1900 °C, respectively. These high melting points help avoid atomic interdiffusion between MgF_2_ and Cr at moderate temperatures during device operation.

**Figure 2:**
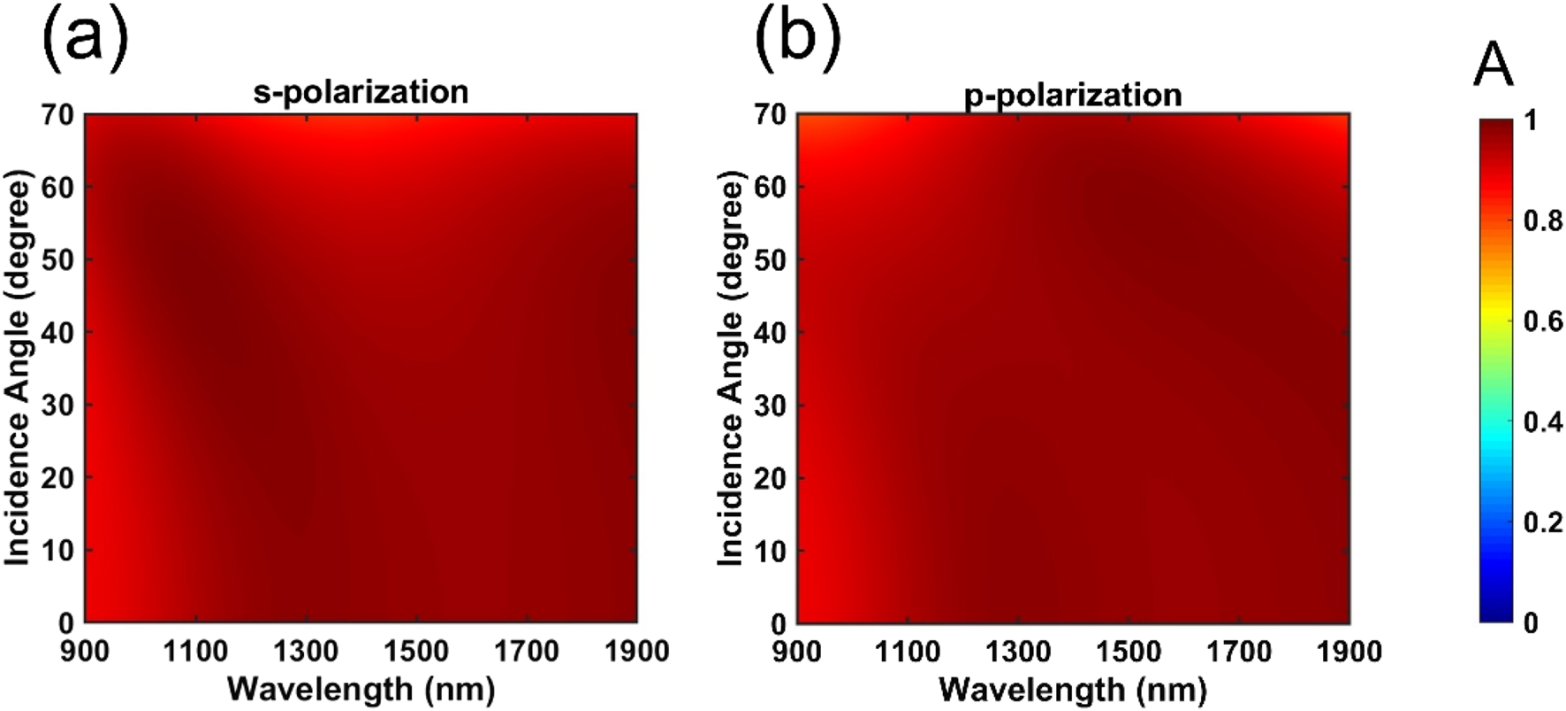
Calculated absorbance contour plots for a perfect broadband absorber with the optimal configuration, t_t_ = 230 nm, t_c_ = 7 nm, and t_d_ = 180 nm. The plots are shown as a function of the wavelength of (**a**) s-polarized and (**b**) p-polarized light with incidence angles ranging from 0° to 70°. The color bar indicates the amount of the absorbance.

**Figure 3:**
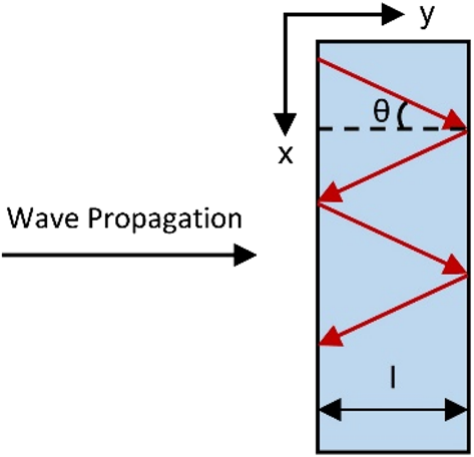
Schematic figure of Fabry–Perot resonance. Wave propagates along y direction. θ is the incidence angle.

As for the design of this layered structure, there appear three key parameters to fundamentally govern the absorption behaviors- t_t_, t_d_ as well as t_c_, denoting the thicknesses of the top MgF_2_, middle MgF_2_ and Cr layers, respectively. We calculated the demanded absorption spectra by using the TMM, sweeping the thicknesses of each layer from 0 to 1000 nm, in particular for t_t_ and t_d_. The optimal absorption was achieved with the layer thicknesses of 230 nm (t_t_), 7 nm (t_c_), and 180 nm (t_d_). Besides, the bottom Cr layer was 100 nm thick, which is much thicker than the skin depth to block the transmittance. With this facile configuration, the absorption efficiency of this PA device was over 90% for both s-polarized and p-polarized light in the wavelength range of 900 to 1900 nm at normal incidence, as shown in Fig. 2. Note that the multilayered structure also presented an impressive tolerance for oblique incidence angles due to the non-resonant structure. The calculated absorbance contour plots for s-polarized and p-polarized light are shown in Fig. 2a,b, respectively. Clearly, we observed that for both polarizations, the absorbance was greater than 80% within wide incidence angles of − 70° to + 70°. Besides, the absorption spectrum in Fig. 2a,b show the blue-shifting of the “main” resonance peak, i.e., the “most red” portion, when the incidence angle increases. This shifting phenomenon can be explained by Fabry–Perot cavity modes in the multilayer structure, as illustrated in Fig. 3. According to the Fabry–Perot condition, one can predict the resonance wavelength by Eq. (1),

1 $$ k_{y} *l = k*\cos \left( \theta \right){*}l = \frac{2\pi }{\lambda }*\cos \left( \theta \right)*l = 2{\text{n}}\pi = {\text{constant,}} $$

where k is the wavevector of incidence wave, k_y_ is the wavevector of incidence wave in y direction, θ is the incidence angle. Therefore, as θ increases (cos(θ) decreases), λ should be blue-shifted to maintain the constant. So, we can observe this blue shift phenomenon in Fig. 2a,b.

**Figure 4:**
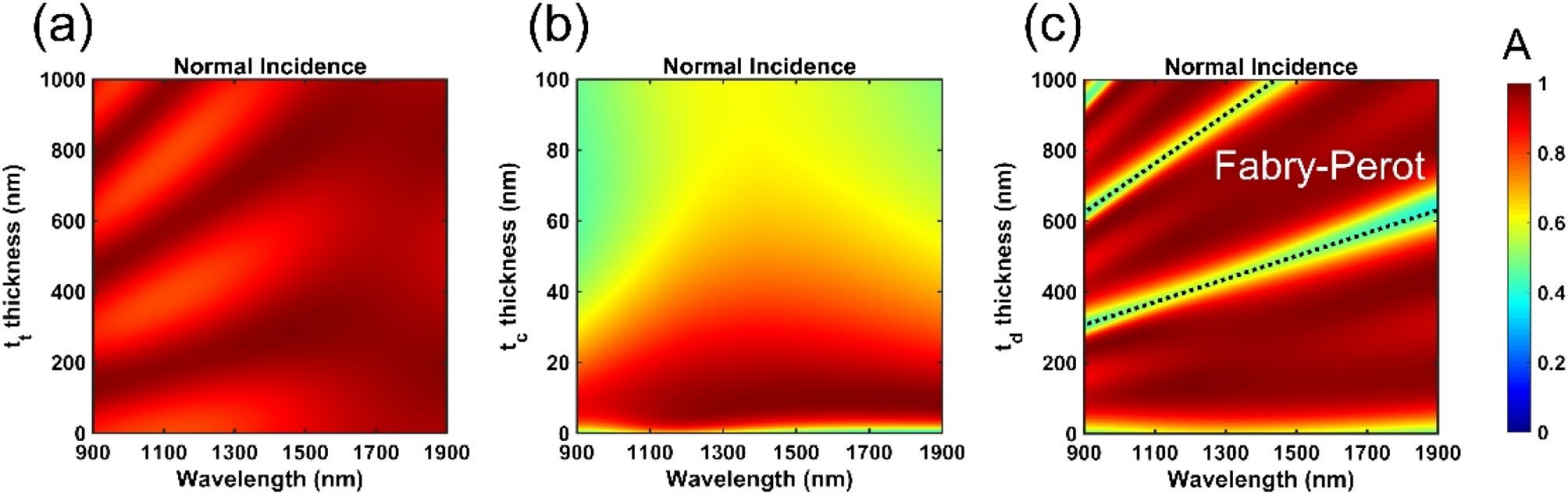
Absorbance spectra of sweeping three layer thicknesses t_t_, t_c_ and t_d_, respectively, under normally incident light. (**a**) Absorbance spectrum plotted as a function of wavelength and the thickness of the top dielectric layer (t_t_) when t_d_ = 180 nm and t_c_ = 7 nm. (**b**) Absorbance spectrum plotted as a function of wavelength and the thickness of the metallic layer (t_c_) when t_t_ = 230 nm and t_d_ = 180 nm. (**c**) Absorbance spectrum plotted as a function of wavelength and the thickness of the middle dielectric layer (t_d_) when t_t_ = 230 nm and t_c_ = 7 nm. The color bar indicates the amount of the absorbance.

We scrutinized the contribution and mechanism of three respective layer thicknesses t_t_, t_c_ and t_d_, by using a commercial software package Comsol 5.3a. The results are presented in Fig. 4. Firstly, varying t_t_ (i.e., the thickness of the top MgF_2_ layer) contributed to similar absorbance, in the wavelength range of 900–1900 nm at normal incidence, as displayed in Fig. 4a. Upon oblique incidences, yet, there appeared distinct absorption behaviors of p- and s-polarizations. For example, as the indecent angle was up to 70°, the absorbance of p-polarization remained insensitive to wavelengths, but that of s-polarization fluctuated between 90 and 30% (see the Figure S3a in the Sect. 3 of Supplementary Information). Therefore, we further simulated two extreme thicknesses of 10 and 500 nm, to check the corresponding absorbance spectra and electric amplitude distributions. The absorbance spectra about t_t_ = 10 nm is shown in Figure S4b. Note that at 70° incidence angle, the absorbance of p-polarized light was still over 80%, but the absorbance of the s-polarized light dropped below 60%. Besides, the field amplitude distribution and absorbance energy in the direction of propagation are plotted as functions of the wavelength at 70° incidence angles for s-polarization in Figure S4c,d. A large portion of the electric field located outside the structure, because the field was blocked and reflected by the top Cr layer after the wave penetrated through a 10 nm thin top MgF_2_ layer. Such strong reflection caused the PAs absorbing s-polarized light poorly. We then increased the thickness of the top MgF_2_ layer to 500 nm (see the Figure S5 in the Sect. 3 of Supplementary Information). In this case, though the thickness of the top MgF_2_ layer was much thicker than the skin wavelength, the reflection of the s-polarized light was significant because of impedance mismatched, leading to a decrease in absorption.

Next, we examined the thickness of the Cr layer, t_c_, from 0 to 100 nm. The effect of varying t_c_ was straightforward. As manifested in Fig. 4b and S5, if t_c_ was too thick, the electromagnetic wave was strongly reflected; on the other hand, if t_c_ was too thin, the limited absorbing region tarnished the energy absorption efficiency. Finally, we probed the thickness of the interior MgF_2_ layer, t_d_, and found out that t_d_ contributed to the absorption most among three thicknesses. As shown in Fig. 4c and S6, the absorbance surpassed 90% as t_d_ was between 100 and 250 nm. In particular, the absorbance even reached nearly perfect efficiency at a t_d_ value of 180 nm. Beyond the range of 100–250 nm, the excellent absorption performance started to fade away. For instance, once t_d_ was below 100 nm, the adjacent Cr layers were too close to one another and then collectively interacted with the electromagnetic wave to function as one thick Cr layer, such that a significant amount of light was reflected, resulting in poor absorption (see the Figure S8 in the Sect. 5 of Supplementary Information). In contrast, as t_d_ exceeded 250 nm, there formed a Fabry–Perot cavity in the middle dielectric layer, so that the maximum local field existed in the less lossy MgF_2_ layer instead of the lossy Cr layer (see the Figure S9 in the Sect. 5 of Supplementary Information). As a consequence, the absorption remarkably degraded, which was clearly indicated by the black dashed lines in Fig. 4c.

**Figure 5:**
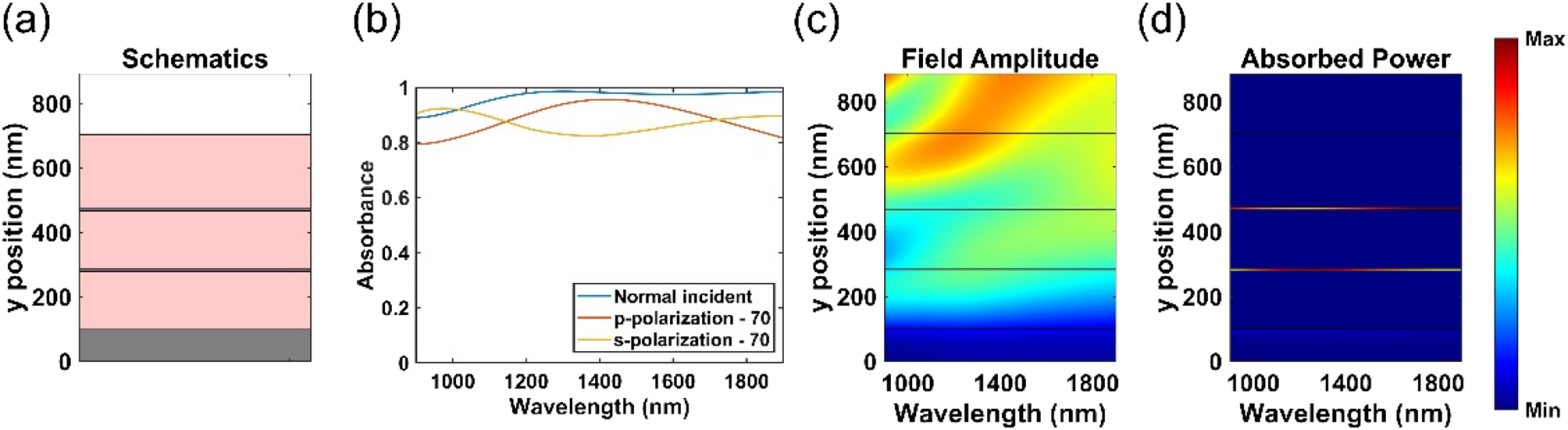
Optimized design. (**a**) Schematic figure. The multilayer along the y-axis respectively correspond to Cr (0–100 nm), MgF_2_ (100–280 nm), Cr (280–287 nm), MgF_2_ (287–467 nm), Cr (467–474 nm), and MgF_2_ (474–704 nm). (**b**) Calculated absorbance spectra obtained under s-polarized and p-polarized light at normal and oblique incidence angles. (**c**) Field amplitude and (**d**) absorbed power along the direction of propagation as a function of the wavelength at normal incidence.

Based on the aforementioned contribution and mechanism, we conducted full factorial sweeping to optimize this multilayered structure. Three layer thicknesses of this optimized non-resonant perfect absorber are t_t_ = 230 nm, t_c_ = 7 nm, and t_d_ = 180 nm, illustrated in Fig. 5a. In this case, we can achieve both higher than 90% absorbance under normal incidence and higher than 80% absorbance under up to 70° incidence angle within 900–1900 nm wavelength range. As evidenced in Fig. 5c, the top Cr layer did not block the electric field, so the incident wave can penetrate into the multilayered structure and then decreased gradually; besides, no Fabry–Perot cavity formed in the middle MgF_2_ layer to deteriorate the absorption. The absorbed power in a broad band of 900–1900 nm was presented in Fig. 5d, which manifested that the absorption was vigorous and uniform in the top Cr layer within the entire working wavelength range. Notice that we also observed that our PA can substantially tolerate inaccurate fabrication of thicknesses in the range of t_t_ (200–250 nm), t_c_ (5–13 nm) and t_d_ (160–190 nm). For example, our device still provided superior absorbance higher than 80%, within a broad wavelength range of 900 to 1900 nm for the normal and oblique incidences, as shown in Fig. 4 and Figure S3, S6, S7 in the Supplementary Information. Such thickness tolerance offers a further benefit for practical device implementation.

## Sample fabrication and experimental verification

To fabricate the designed multilayered structure, a silicon (Si) wafer was first cut into small pieces. The samples were soaked in piranha solution for 15 min to remove the organic contamination, and then the native oxide layer on the Si was removed by using hydrofluoric acid. Next, Cr and MgF_2_ layers with the desired thicknesses were deposited by using an electron beam (EB) evaporation process, in which the deposition rates of Cr and MgF_2_ approximated 1 and 5 Å/s, respectively, and the chamber pressure was held below 4 × 10^−6^ torr throughout the deposition process. First, we deposit 100-nm Cr and MgF_2_ at a constant rate and measure the real thickness of the deposited thin film using atomic force microscope, which makes sure to deposit the desired thickness. The SEM image is shown in Fig. 1b. To measure the intensity of 900–2000-nm light reflected by the structure in the normal direction, we used a Hyperion 3000 Fourier-transform infrared (FTIR) microscope with a 15 × objective (Bruker, Billerica, MA, USA). The FTIR spectra were collected in reflection mode using a tungsten lamp as a light source and an InGaAs diode for detection. The angular response of the perfect absorber to p- and s-polarized light incident at angles of 40°, 60°, and 70° (θ) was evaluated using a commercially available Ellipsometer (see the Figure S10 in the Sect. 6 of Supplementary Information). All of the measured reflectance values were normalized to the reflectance of a thick Au-coated sample, which was near 100% reflectance in our desired frequency range.

**Figure 6:**
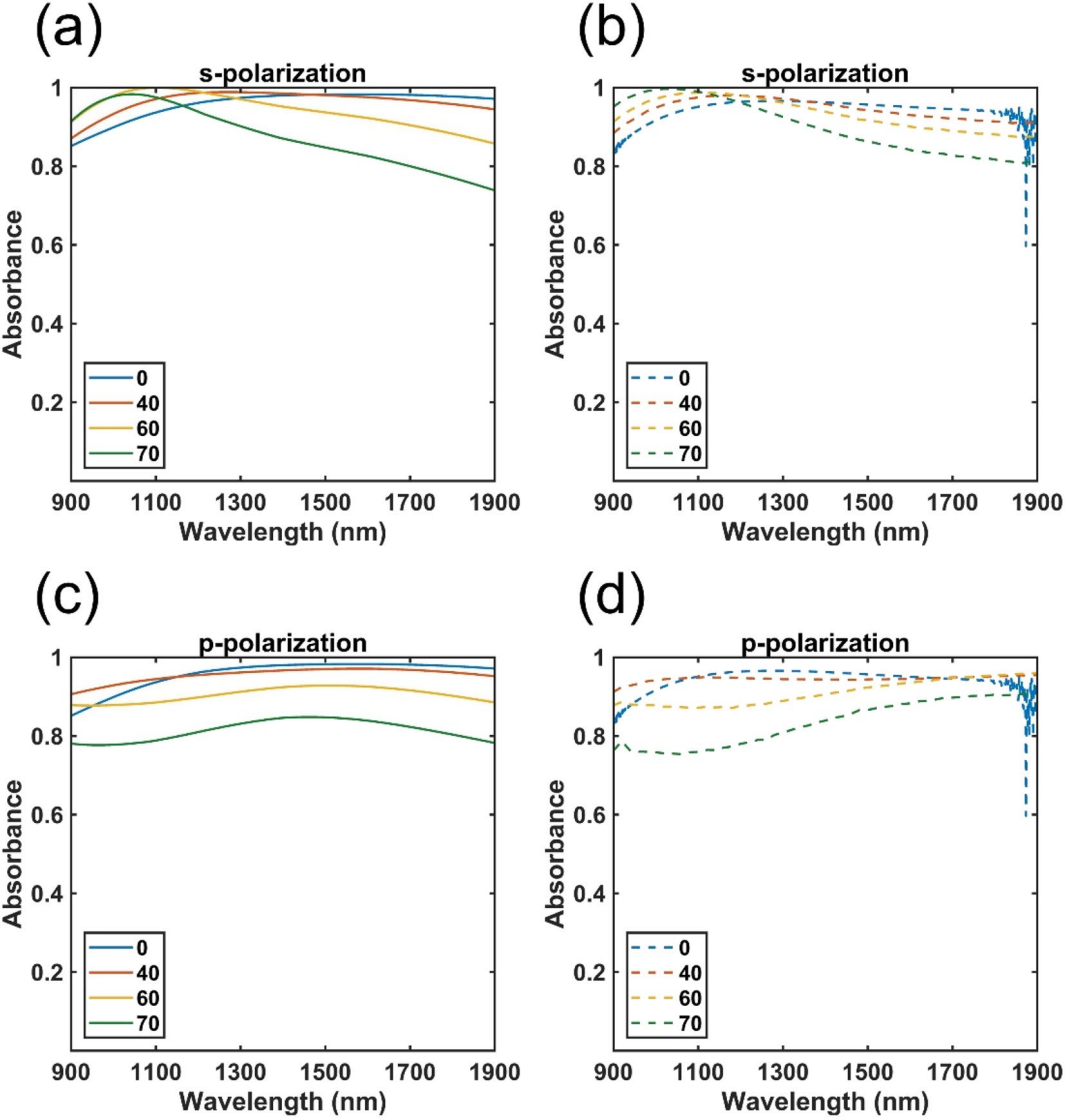
Absorbance spectra of perfect absorbers under s- and p-polarized light at various incidence angles with the configuration, t_t_ = 230 nm, t_c_ = 13 nm, and t_d_ = 180 nm. (**a**) Calculated and (**b**) experimental results under s-polarization. (**c**) Calculated and (**d**) experimental results under p-polarization. Normal incidence and oblique angle was measured by FTIR and Ellipsometer respectively.

The measured absorbance results are shown in Fig. 6a,b. For the experimental results, the thickness of Cr is around 13 nm but not 7 nm due to the limitation of E-gun deposition process. For the normal incident cases, there are some noise around 1900 nm due to the limitation of FTIR detector. Note that to maximize the absorbance, we need to minimize both reflectance and transmittance, as depicted by the following equation,

$$ A = 1 - R - T = 1 - R $$

where A, R, and T represent absorbance, reflectance, and transmittance, respectively. The bottom Cr layer was 100 nm thick, which is much thicker than the skin depth to block the transmittance. So, absorbance is the complementary part of the reflectance. For conventional resonant PAs, the reflectance can be shut down by the resonance which will form antiparallel currents within upper and bottom metallic layer. However, such configuration only function at specific wavelength and small oblique incidence angle limitation. In contrast, here the fabricated non-resonance multilayer structure efficiently absorbed light by the intrinsic loss of the materials. Most importantly, the experimental results well agree with the calculated results, as shown in Fig. 6c,d. The experimental absorption is even higher than the calculation absorption in some certain cases. That is because the litter rough on the top MgF_2_ layer which will decrease the amount of reflectance. For both experimental and calculated absorption approached or exceeded 80%, even when the oblique incidence angle up to 70°. To the best of our knowledge, this is the broadest absorption range reported for a multilayered device fabricated without the use of lithography. The performance of this absorber was not only with great efficiency, but also free from polarization, oblique incidence angles up to 70°, and lithographic process.

## Conclusions

In this study, we presented a non-resonant perfect absorber (PA), which is composed of six pile-stacking layers of magnesium fluoride (MgF_2_) and chromium (Cr). The key contributions and mechanisms of MgF_2_ are to match the impedance for minimizing the reflectance and to act as spacers between metal layers; meanwhile, Cr, not only as a ground layer shuts own the transmittance, but also as middle layers play a key role of absorbing incident wave because of its great intrinsic loss. By optimizing the thickness of individual layers, we demonstrated excellent absorbance over 90% at normal incidence. Owing to its non-resonance nature, this PA possesses further advantages beyond conventional resonance-based perfect PAs: a broad operation range of 900–1900 nm, polarization insensitivity to both s- and p-lights, wide allowed incident angles up to 70°, cost-effective and inaccuracy-tolerate fabrication processes. In addition, comparing with non-resonance-type absorbers such as carbon nanotubes, our PA is ultrathin. These aforementioned features of our PA were consistently confirmed in experimental measurements, analytical calculation and numerical simulation, paving a way toward practical and instant applications.

## Supplementary Information

Supplementary Information
