# Supplementary material for: Ultra-broadband, lithography-free, omnidirectional, and polarization-insensitive perfect absorber
Source: Sci Rep. 2021 Mar 4;11:5173. doi: 10.1038/s41598-021-84889-0 (PMC7933432; doi:10.1038/s41598-021-84889-0)
Supplement: Supplementary file 1 — Supplementary Information [file 41598_2021_84889_MOESM1_ESM.docx]

Supplementary Information

Ultra-broadband, lithography-free, omnidirectional, and polarization-insensitive perfect absorber

Tse-An Chen^1^, Meng-Ju Yub^2^, Yu-Jung Lu^2^, and Ta-Jen Yen^*1^

^1^ Department of Materials Science and Engineering, National Tsing Hua University, No. 101, Section 2, Kuang-Fu Road, Hsinchu, Taiwan 30013, R.O.C.

^2^ Research Center for Applied Sciences, Academia Sinica, 128 Sec. 2, Academia Rd., Nankang, Taipei City, Taiwan

*tjyen@mx.nthu.edu.tw

**Section 1. Comparison between MgF_2_ and Al_2_O_3_**

**
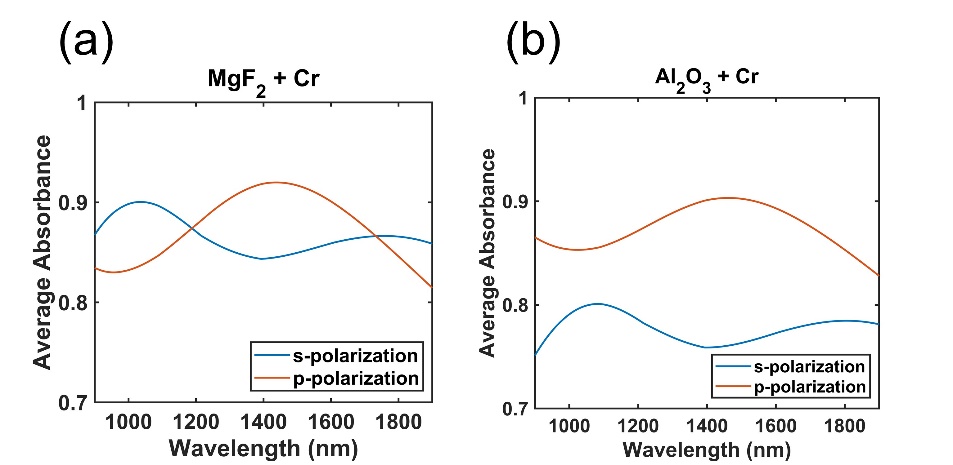
**

In order to help audience to understand the different of the absorption performance between low and high refractive index material. We selected two material to do the comparison. One was MgF_2_, which possessed lower refractive index. The other one was Al_2_O_3_, which had higher refractive index comparing to the MgF_2_. The optimal parameters for Al_2_O_3_ + Cr are t_t_ = 180 nm, t_c_ = 10 nm, and t_d_ =140 nm. The optimal parameters for MgF_2_ + Cr are t_t_ = 230 nm, t_c_ = 7 nm, and t_d_ =180 nm. Next, we calculated the average absorption by detuning the parameters t_t_ and t_d_ for ± 40 nm. Take MgF_2_ + Cr as an example, t_t_ = 190, 230, 270 nm and t_d_ =140 nm, 180nm, 220nm. Therefore, we had nine combination sets in total. Then, we calculated the average absorbance of above nine sets. The results are shown in Figure S1. The average absorbance performance of MgF_2_ + Cr is slightly better than Al_2_O_3_ + Cr due to the lower refractive index of MgF_2_, which could decrease the “Fresnel reflections” to air.

**Section 2. Optical Properties of Cr, Au, Ag and MgF_2_**

**
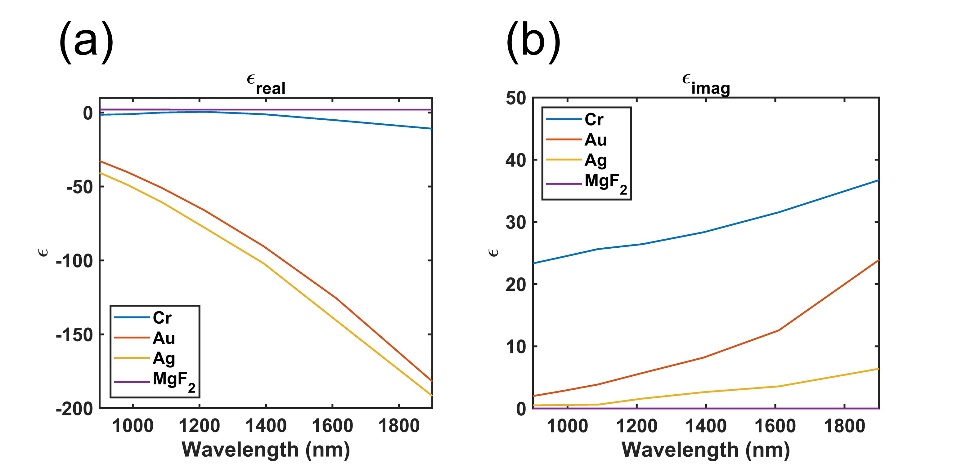
**

Figure S2. Optical properties of Cr^S1^, Au^S2^, Ag^S2^ and MgF_2_^S3^ within working wavelength range. (a) Real part of the permittivity. (b) Imaginary part of the permittivity

**Section 3. Absorption performance on the geometry of sweeping thickness of top dielectric layer (t_t_)**

**
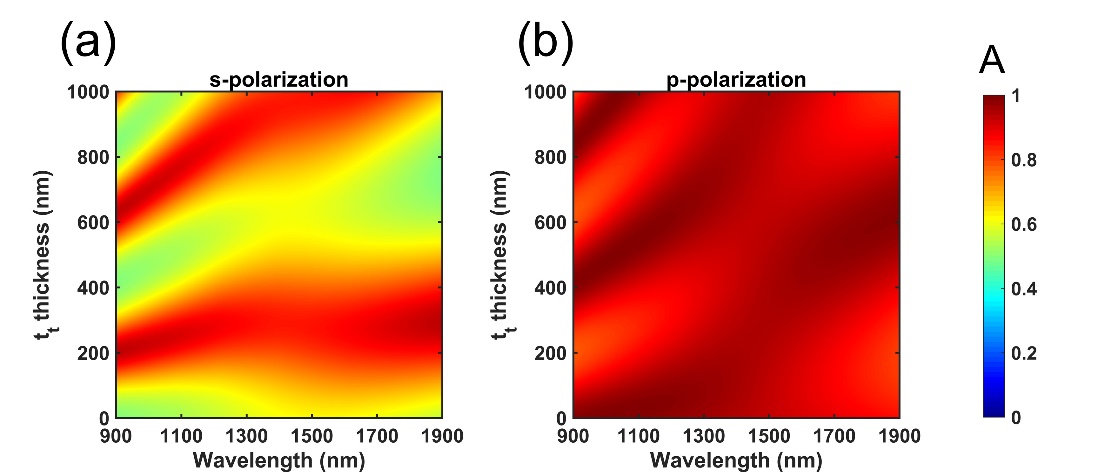
**

Figure S3. Absorbance spectrum plotted as a function of wavelength and the thickness of the top dielectric layer (t_t_) when t_d_ = 180 nm and t_c_ = 7 nm under 70° incidence angle in (a) s- and (b) p-polarizations.


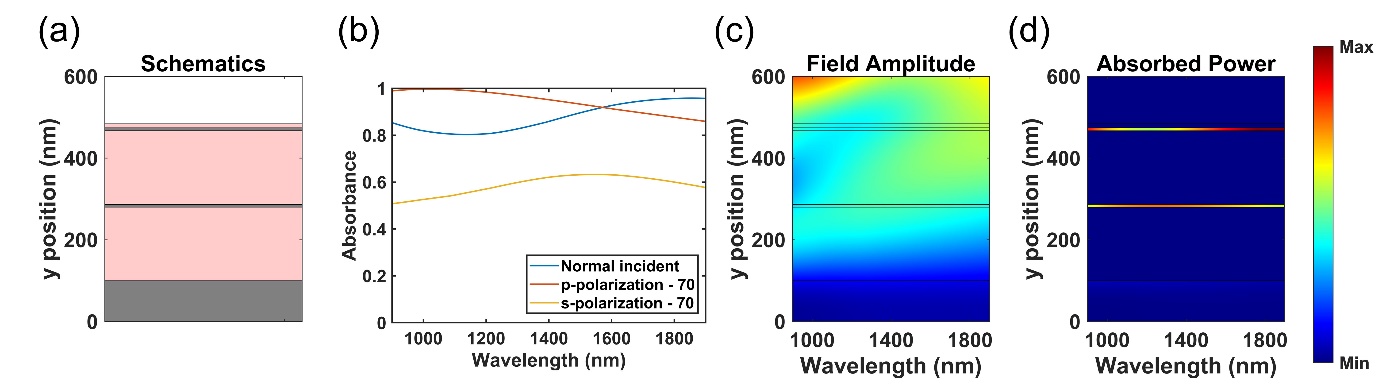


Figure S4. Simulation results with t_t_ = 10 nm, t_c_ = 7 nm and t_d_ = 180 nm. (a) Schematic figure. The multilayer along y-axis is respectively, Cr (0-100 nm), MgF_2_ (100-280 nm), Cr (280-287 nm), MgF_2_ (287-467 nm), Cr (467-474 nm), MgF_2_ (474-484 nm). (b) Calculated absorbance spectra obtained under s-polarized and p-polarized light at normal and oblique incidence angles. (c) Field amplitude and (d) absorbed power along the direction of propagation as a function of the wavelength at 70 incidence angles under s-polarization.


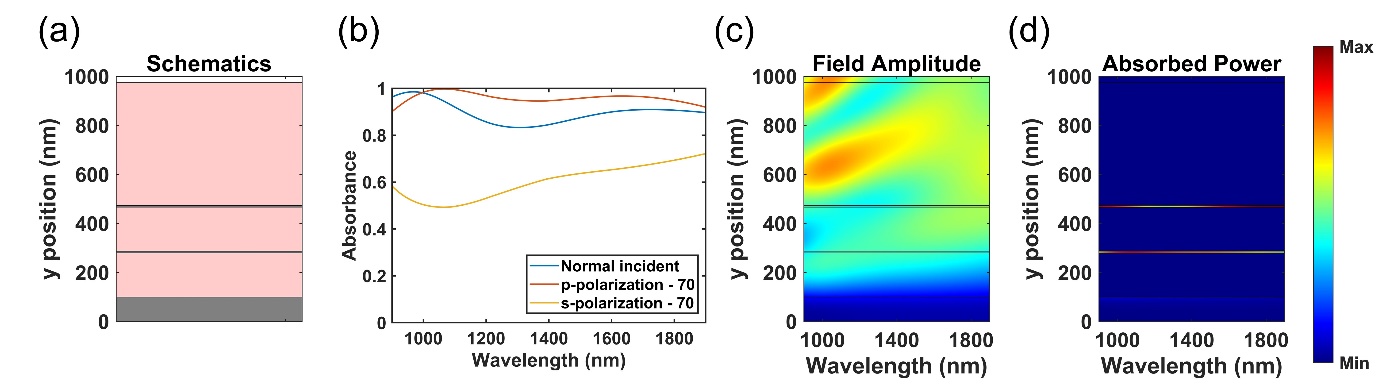


Figure S5. Simulation results with t_t_ = 500 nm, t_c_ = 7 nm and t_d_ = 180 nm. (a) Schematic figure. The multilayer along y-axis is respectively, Cr (0-100 nm), MgF_2_ (100-280 nm), Cr (280-287 nm), MgF_2_ (287-467 nm), Cr (467-474 nm), MgF_2_ (474-974 nm). (b) Calculated absorbance spectra obtained under s-polarized and p-polarized light at normal and oblique incidence angles. (c) Field amplitude and (d) absorbed power along the direction of propagation as a function of the wavelength at 70 incidence angles under s-polarization.

**Section 4. Absorption performance on the geometry of sweeping thickness of metallic layer (t_c_)**


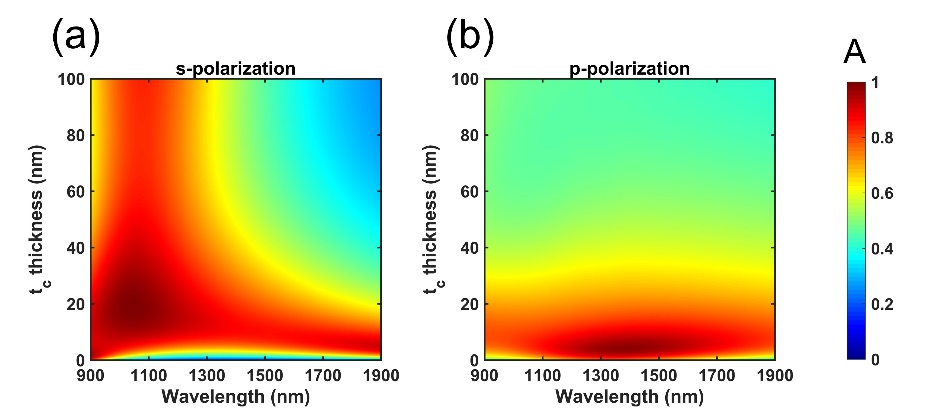


Figure S6. Absorbance spectrum plotted as a function of wavelength and the thickness of the metallic layer (t_c_) when t_t_ = 230 nm and t_d_ = 180 nm under 70° incidence angle in (a) s- and (b) p-polarizations.

**Section 5. Absorption performance on the geometry of sweeping thickness of middle dielectric layer (t_d_)**


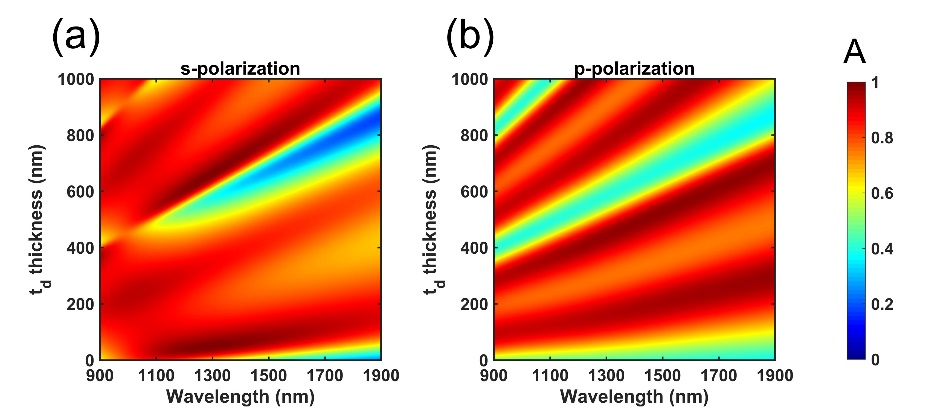


Figure S7. Absorbance spectrum plotted as a function of wavelength and the thickness of the middle dielectric layer (t_d_) when t_t_ = 230 nm and t_c_ = 7 nm under 70° incidence angle in (a) s- and (b) p-polarizations.


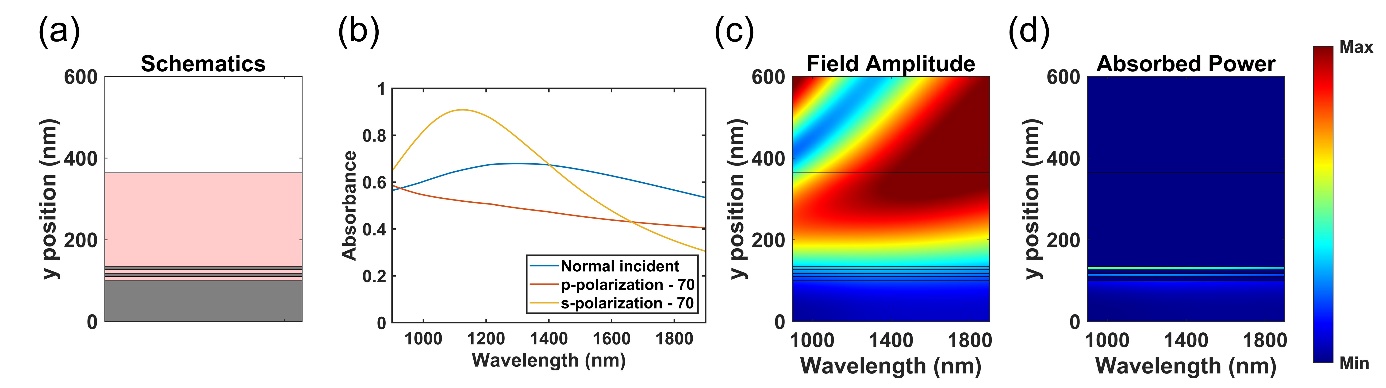


Figure S8. Simulation results with t_t_ = 230 nm, t_c_ = 7 nm and t_d_ = 10 nm. (a) Schematic figure. The multilayer along y-axis is respectively, Cr (0-100 nm), MgF_2_ (100-110 nm), Cr (110-117 nm), MgF_2_ (117-127 nm), Cr (127-134 nm), MgF_2_ (134-364 nm). (b) Calculated absorbance spectra obtained under s-polarized and p-polarized light at normal and oblique incidence angles. (c) Field amplitude and (d) absorbed power along the direction of propagation as a function of the wavelength at normal incidence.


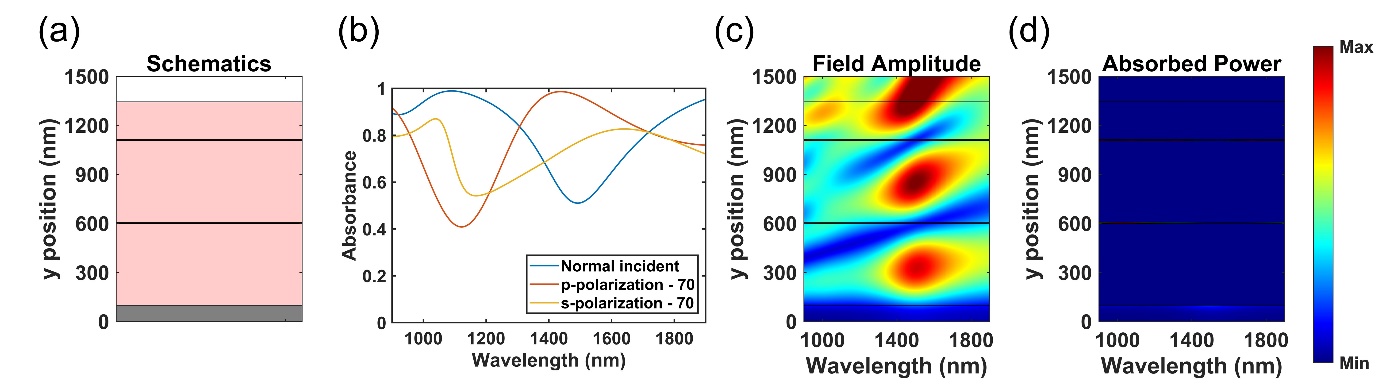


Figure S9. Simulation results with t_t_ = 230 nm, t_c_ = 7 nm and t_d_ = 500 nm. (a) Schematic figure. The multilayer along y-axis is respectively, Cr (0-100 nm), MgF_2_ (100-600 nm), Cr (600-607 nm), MgF_2_ (607-1107 nm), Cr (1107-1114 nm), MgF_2_ (1114-1344 nm). (b) Calculated absorbance spectra obtained under s-polarized and p-polarized light at normal and oblique incidence angles. (c) Field amplitude and (d) absorbed power along the direction of propagation as a function of the wavelength at normal incidence.

**Section 6. Experimental setup**


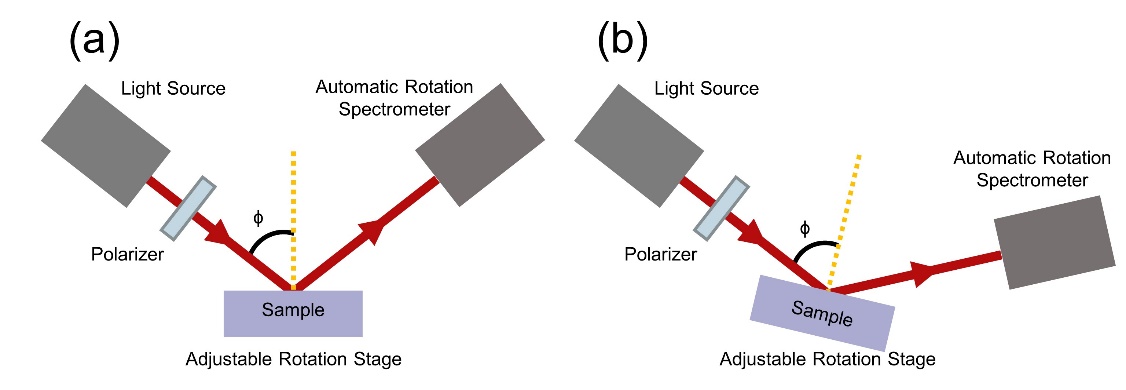


Figure S10. Illustration of optical setup of Ellipsometer.

**References**

S1 Johnson, P. B. & Christy, R. W. Optical constants of transition metals: Ti, V, Cr, Mn, Fe, Co, Ni, and Pd. *Physical Review B* **9**, 5056-5070, doi:10.1103/PhysRevB.9.5056 (1974).

S2 Johnson, P. B. & Christy, R. W. Optical Constants of the Noble Metals. *Physical Review B* **6**, 4370-4379, doi:10.1103/PhysRevB.6.4370 (1972).

S3 Self-consistent optical constants of MgF2, LaF3, and CeF3 films. *Opt. Mater. Express* **7**, 989-1006, doi:10.1364/OME.7.000989 (2017).
